# ADP-heptose attenuates *Helicobacter pylori*-induced dendritic cell activation

**DOI:** 10.1080/19490976.2024.2402543

**Published:** 2024-09-17

**Authors:** Theresa Neuper, Tobias Frauenlob, Hieu-Hoa Dang, Peter W. Krenn, Gernot Posselt, Christof Regl, Nikolaus Fortelny, Veronika Schäpertöns, Michael S. Unger, Gunda Üblagger, Daniel Neureiter, Iris Mühlbacher, Michael Weitzendorfer, Franz Singhartinger, Klaus Emmanuel, Christian G. Huber, Silja Wessler, Fritz Aberger, Jutta Horejs-Hoeck

**Affiliations:** a Department of Biosciences and Medical Biology, Paris-Lodron University Salzburg, Salzburg, Austria; b Center for Tumor Biology and Immunology, Paris-Lodron University Salzburg, Salzburg, Austria; c Cancer Cluster Salzburg, Salzburg, Austria; d Institute of Pathology, Uniklinikum Salzburg, Salzburg, Austria; e Department of General, Visceral and Thoracic Surgery, Paracelsus Medical University/Salzburger Landeskliniken (SALK), Salzburg, Austria

**Keywords:** Dendritic cells, H. pylori, ADP-heptose, type I IFN, TLR2

## Abstract

Sophisticated immune evasion strategies enable *Helicobacter pylori (H. pylori)* to colonize the gastric mucosa of approximately half of the world’s population. Persistent infection and the resulting chronic inflammation are a major cause of gastric cancer. To understand the intricate interplay between *H. pylori* and host immunity, spatial profiling was used to monitor immune cells in *H. pylori* infected gastric tissue. Dendritic cell (DC) and T cell phenotypes were further investigated in gastric organoid/immune cell co-cultures and mechanistic insights were acquired by proteomics of human DCs. Here, we show that ADP-heptose, a bacterial metabolite originally reported to act as a bona fide PAMP, reduces *H. pylori*-induced DC maturation and subsequent T cell responses. Mechanistically, we report that *H. pylori* uptake and subsequent DC activation by an ADP-heptose deficient *H. pylori* strain depends on TLR2. Moreover, ADP-heptose attenuates full-fledged activation of primary human DCs in the context of *H. pylori* infection by impairing type I IFN signaling. This study reveals that ADP-heptose mitigates host immunity during *H. pylori* infection.

## Introduction

*Helicobacter pylori* (*H. pylori*) is one of the most successful human pathogens, infecting approximately 50% of the world’s population. This unique pathogen ensures its own survival within the stomach by perfectly adapting to and shaping its niche.^1^ Chronic infection with *H. pylori* is asymptomatic in most cases, but 10–15% of infected individuals develop gastric ulcers and 1–3% stomach cancer,^2^ which is the third leading cause of cancer-related deaths worldwide.^3,4^ Accordingly, the International Agency for Research on Cancer has declared *H. pylori* to be a class 1 (definite) carcinogen.^5^ Although, *H. pylori* is masterful at evading immune recognition and manipulating host immune responses,^6^ infection still results in potent inflammatory responses in cells of the innate and adaptive immune system.^7,8^

A key feature of the innate immune system’s rapid response to infection is its ability to recognize conserved pathogen-associated molecular patterns (PAMPs).^9,10^ In this context, metabolic intermediates of lipopolysaccharide (LPS) biosynthesis are attracting intense interest as they have been identified as novel PAMPs of various Gram-negative bacteria, including *H. pylori*. The immunostimulatory capacities of these metabolites were initially described in studies involving infections with *Neisseria gonorrhoeae, Neisseria meningitidis*, *Shigella flexneri, Salmonella enterica serovar* Typhimurium or *H. pylori*, showing that NF-κB activation as well as secretion of pro-inflammatory cytokines is induced by HMP (D-*glycero*-β-D-*manno*-heptose 1-monophosphate) or HBP (D-*glycero*-β-D-*manno*-heptose 1-biphosphate).^11–16^ However, soon afterward, in 2018, it was shown that only one heptose metabolite, namely ADP-heptose (ADP-*glycero*-β-D-*manno*-heptose), could effectively enter the host cell cytosol to robustly activate inflammatory responses. HBP, in contrast, was found to require conversion to ADP-heptose-7-phosphate by host adenylyltransferase to elicit a pro-inflammatory response. Although HBP indeed triggers inflammatory signals, the effects of HBP are modest in comparison to those induced by ADP-heptose. Identification of a physical interaction of ADP-heptose with the N-terminal domain of the serine/threonine-specific kinase alpha kinase 1 (ALPK1) led to assignment of the ADP-heptose/ALPK1 complex to the PAMP – pattern recognition receptor (PRR) family.^17–19^ Recognition of ADP-heptose by its cognate receptor results in phosphorylation of TRAF-interacting protein with forkhead-associated domain (TIFA) to trigger its oligomerization, leading to the recruitment of various other downstream interaction partners to form a multiprotein-complex termed the TIFAsome.^16^ Based on detailed mechanistic studies it is now well-established that TIFA forms a complex with TNF receptor-associated factor (TRAF) 6 or TRAF2, which in turn mediates the induction of classical or alternative NF-κB signaling, respectively, and promotes the secretion of CXCL8 in epithelial cells.^11,14,16,20^ Moreover, in *H. pylori-*infected epithelial cells the ALPK1/TIFA/NF-κB axis has been shown to induce DNA damage via the induction of R-loop-associated replication stress.^21^ To date, most studies conferring PAMP status to ADP-heptose have relied on epithelial cell models. Interestingly, at the time that ALPK1 was identified as the cognate receptor of ADP-heptose, Ryzhakov and colleagues reported that *Alpk1*^*−/−*^ mice are more susceptible to *Helicobacter hepaticus*-induced colitis and that this condition is mainly driven by Alpk1 deficiency in hematopoietic cells. In particular, they identified that *Alpk1*^*−/−*^ macrophages produce increased amounts of inflammatory cytokines, including Interleukin 12 (IL-12), upon *H. hepaticus* infection and thus concluded that ALPK1 suppresses IL-12-mediated, Th1-driven intestinal inflammation.^22^ Their study suggests that in hematopoietic cells, ALPK1 signaling attenuates rather than promotes inflammatory responses.

To further our understanding of the role of ADP-heptose beyond the gastric epithelium, we aimed to investigate its capacity to activate human dendritic cells (DCs), the most adept antigen-presenting cells, by using *H. pylori* infection as a model system. As sentinels of the immune system, DCs are not only well-equipped with a plethora of PRRs, but they are also essential for maintaining the delicate balance between activating potent inflammatory responses and inducing tolerance in different pathological settings. However, activation of DCs in the context of *H. pylori* infection is controversial, since several studies have concluded that *H. pylori* induces tolerogenic DCs,^23–25^ while others clearly show that *H. pylori* infection results in the secretion of a variety of pro-inflammatory mediators and the expression of co-stimulatory surface molecules.^7,8,26,27^ In this study, we show that DCs are recruited in high numbers into *H. pylori-*infected gastric mucosa, and that they are potently activated upon *H. pylori* infection in an in vitro model of the gastric epithelial environment. Moreover, we report that ADP-heptose barely activates human DCs compared to the well-studied bacterial PAMP LPS from *E. coli*. Instead, and even more intriguingly, ADP-heptose potently suppresses the Th-1-associated inflammatory response in DCs. Our mechanistic investigations revealed that TLR2 promotes *H. pylori* uptake and the enhanced CD40 expression upon infection with the ADP-heptose deficient *H. pylori* mutant. Moreover, ADP-heptose dampens *H. pylori-*induced type I interferon (IFN) signaling, resulting in decreased IL-12p70 secretion upon *H. pylori* infection. Thus, rather than initiating potent inflammatory responses in professional antigen-presenting cells similar to other bona fide PAMPs, ADP-heptose appears to exert an immune-dampening effect on human DCs and subsequent T cell responses.

## Materials and methods

### Isolation of primary human immune cells from peripheral blood

This study was conducted in accordance with the approved guidelines of the World Medical Association’s Declaration of Helsinki and the guidelines of the Ethics Committee of the Province of Salzburg. No additional form of consent is required from anonymized blood donors for scientific use of leukapheresis products according to Austrian national regulations.

Human primary CD1c^+^ (BDCA1^+^) DCs were isolated from fresh buffy coats obtained from the Blood Bank Salzburg. Briefly, PBMCs were isolated from human leukapheresis products via gradient density centrifugation using HistoPaque-1077 (Sigma-Aldrich 10,771). Thereafter, DCs were isolated via magnetic cell sorting according to the manufacturer’s instructions using the Human CD1c (BDCA1)^+^ Dendritic Cell Isolation Kit (Miltenyi Biotec, 130-119-475). DCs were then cultured in RPMI-1640 (Sigma-Aldrich, R0083) supplemented with 10% heat-inactivated fetal calf serum (Biowest, BS-2020-500) and 1% L-glutamine (Sigma-Aldrich, G7513). After isolation, DCs were incubated between 1 and 4 h at 37°C until further use. For qPCR and co-culture experiments, DCs were seeded at a density of 10^5^ cells/mL, and for flow cytometry and Western Blot analysis at 2 × 10^5^ cells/mL. Primary human naïve CD4^+^ T cells or total T cells were isolated from PBMCs as described above via magnetic cell sorting according to the manufacturer’s instructions using a Human Naïve CD4^+^ T Cell Isolation Kit II (Miltenyi Biotec, 130-094-131) or Human Pan T Cell Isolation Kit (Miltenyi Biotec, 130-096-535), respectively. T cells were subsequently incubated with DCs as described below.

### DC/T cell co-culture

DCs were infected with *H. pylori* for 18 h before Penicillin and Streptomycin (1×, Sigma-Aldrich, P4333) was added to the medium, to avoid direct infection of naïve T cells. After another 6 h of incubation, freshly isolated allogenic naïve CD4^+^ T cells or total T cells were added to the DC culture at a ratio of 1:10 (10^5^ DCs: 10^6^ T cells). Co-culture was performed for 6 days, thereafter cytokine and surface marker expression of T cells was analyzed by flow cytometry.

### Bacterial culture and infection

*Helicobacter pylori* P12 and its isogenic mutant (ΔrfaE)^16^ were cultivated under microaerophilic conditions on GC agar plates containing 10% horse serum (Biowest, S0910) and selective antibiotics (Kanamycin). Microaerophilic conditions were obtained using Thermo Scientific Oxoid AnaeroJars (Thermo Fisher Scientific) and Oxoid CampyGen 3.5 L sachets (Thermo Fisher Scientifc, CN0025A) and bacteria were cultured over a period of 3 days. One day prior to DC infection, bacteria were re-plated and allowed to grow for another 24 h. *Acinetobacter lwoffii* (DSM 2403) was plated the day before infection of eukaryotic cells on nutrient agar plates and incubated at 37°C and 5°C CO_2_. On the day of infection, cotton buds (Paul Boettger, EH12.1) were used to collect bacteria from plates. Bacteria were resuspended in 1 mL phosphate-buffered saline (PBS, Sigma-Aldrich, D8537) and cell density was determined via spectrophotometric measurement at OD600 (BioPhotometer Plus, Eppendorf). Using an in-house calibration curve bacterial cell numbers were calculated. For all experiments including DCs, an MOI of 5, for infection of mucosoids an MOI of 100 and for PBMCs an MOI of 0.1 was applied.

### Mucosoid culture and infection

Human gastric tissue specimens were provided by the Department of General, Visceral and Thoracic Surgery, Paracelsus Medical University/Salzburger Landeskliniken (SALK) from individuals undergoing gastrectomy or sleeve resection. The corresponding ethics application was approved by the local ethics committee (EK Nr: 1003/2021, Ethikkommission für das Bundesland Salzburg).

Mucosoids were cultured as described previously.^28^ Briefly, approx. 2 × 10^5^ gastric organoid-derived cells were seeded in collagen (Gibco A10644–01, 15 μg/cm^2^-coated 0.4-µm trans-well filters (Millipore, PIHP01250) and cultured for 3 weeks in organoid medium (18.5% v/v Advanced DMEM/F12 (Thermo Fisher Scientific 12,634), 50% v/v Wnt3A conditioned-medium, R-spondin1 25% v/v conditioned medium, 10 mM 4-(2-hydroxyethyl)-1-piperazineethanesulfonic acid (Carl Roth, 9157.1), 2 mM L-glutamine (Biowest, X0550), 2% v/v B27 (Thermo Fisher Scientific 17,504,044), 1% v/v N2 Thermo Fisher Scientific 17,502,048, 20 ng/ml human EGF (Thermo Fisher Scientific, PHG0315), 150 ng/ml human noggin (Peprotech, 120-10C), 150 ng human FGF-10/ml (Peprotech, 100–26), 10 mM nicotinamide (Sigma-Aldrich, N0636), 10 nM human gastrin (Sigma-Aldrich, G9145), 1 μM A83–01 (Sigma, SML0788), 7.5 μM Y-27632 (Sigma-Aldrich, Y0503). Four hours before infection with *H. pylori*, 1.5 × 10^5^ DCs were resuspended in 80 µl of Matrigel (Corning, BDL356231) and DCs were introduced into the sub-chamber compartment of the trans-well inserts. Mucosoids were apically infected with *H. pylori* for 40 h at a multiplicity of infection (MOI) of 100. For downstream experiments, DCs were re-isolated from Matrigel by using Cell Recovery Solution (Corning 354,253) according to manufacturer’s instructions.

### Blocking experiments

Blocking of various targets was performed 20 min prior to infection. Blocking agents were not washed away prior to infection but were present throughout the culture. The following blocking antibodies or inhibitors were used at the indicated concentrations: IFN-Alpha/Beta Receptor Chain 2 blocking antibody (ENZO life sciences, PBL-21385-1) 1 or 5 µg/ml; TAK1 inhibitor Takinib (Selleckchem, S8663) 1–10 µM, Polyclonal anti-hTLR2 (Invivogen, pab-hstlr2) 10 µM, anti-hTLR2 IgA (clone B4H2) (Invivogen, maba2-htlr2-2) 10 µM.

### IFN or ADP-heptose supplementation

DCs were treated with IFNα (Immunotools 11,343,504) and IFNβ (Immunotools 11,343,542) at a concentration of 10 ng/ml each or ADP-heptose (Invivogen, tlrl-adph-l, 2.5 or 25 µg/ml) simultaneously to infection for the indicated time points.

### Luminex assay

Measurement of chemokine and cytokine secretion was performed via bead-based multiplex assay using both an Inflammation 20-Plex Human ProcartaPlex Panel (Thermo Fisher Scientific, EPX200 -12,185-901) or a Cytokine/Chemokine/Growth Factor 45-Plex Human ProcartaPlex Panel (Thermo Fisher Scientific, EPX450 -12,171-901). All following incubation steps are performed on an orbital shaker (450–600 rpm) at indicated temperatures and for specified time periods. For both assays, appropriate amounts of beads were collected in 1.5 ml tube and washed once by adding PBS containing 0.05% Tween-20 (Sigma-Aldrich, P7949) followed by centrifugation. Thereafter, beads were resuspended in Assay Buffer and transferred to 96-well V bottom plates (Greiner BioOne 651,101). Upon addition of standards and samples, the plate was incubated overnight at 4°C. The next day, three washing steps were performed, by adding 150 µl wash buffer/well followed by centrifugation (1650 g, 5 min, 4°C). Supernatants were removed by flipping the plate. Incubation with the biotinylated Detection Antibody for 30 min at room temperature was followed by another three washing steps. Thereafter, streptavidin-labeled PE was added, and the plate was incubated for another 30 min at room temperature. The plate was washed thrice before beads were resuspended in Reading Buffer and wells were measured on a Luminex MagPix instrument (Luminex). The browser-based ProcartaPlex Analyst software was used to analyze the raw data.

### Granzyme B enzyme linked immunosorbent assay (ELISA)

Granzyme B secretion was analyzed using the Granzyme B DuoSet ELISA (R&D Systems, DY2906–05) according to manufacturer’s instructions.

### RNA isolation, cDNA generation and qPCR

Total cellular RNA was extracted from TRI reagent lysates (Sigma-Aldrich, T9424) according to the manufacturer’s instructions. RNA was then reverse-transcribed using RevertAid H Minus M-MuLV reverse transcriptase (Thermo Fisher Scientific, EP0452). mRNA expression levels of genes of interest were then determined by quantitative real-time PCR on a Rotor Gene 3000 device (Corbett Research) utilizing Luna Universal qPCR Master Mix (New England BioLabs, M3003L). Relative mRNA expression was determined by normalizing the mRNA expression value of the gene of interest with the expression level of a housekeeping gene (RPLP0). Relative mRNA expression was calculated as 2^−ΔCt^, with ΔCt representing the difference between cycle threshold (Ct) of the gene of interest minus the Ct of the housekeeping gene. Specificity of the primers was continuously monitored by melting curve analysis. IFNA2 fwd: 5′-gtgctcagctgcaagtcaag−3′, IFNA2 rev: 5′-tctgctggatcatctcatgg−3′, ISG15 fwd: 5′-catctttgccagtacaggag−3′, ISG15 rev: 5′-tgatctgcgccttcagctct−3′, CCL3 fwd: 5′-tgcaaccagttctctgcatc−3′, CCL3 rev: 5′-ctcgtctcaaagtagtcagc−3′, IL1B fwd: 5′-gtacctgagctcgccagtga−3′, IL1B rev: 5′-tcggagattcgtagctggatg−3′, IL10 fwd: 5′-agggcacccagtctgagaaca− 3′, IL10 rev: 5′-cggccttgctcttgttttcac−3′, CCL4 fwd: 5′-tcattgctactgccctctgc−3‘, CCL4 rev: 5′-tcgggtgacaaagacgactg−3′, CXCL8 fwd: 5′-ccaggaagaaaccaccggaag−3′, CXCL8 rev: 5′-tggtccactctcaatcactctcag−3′, CXCL1 fwd: 5′-cactgctgctcctgctcctggtag−3′, CXCL1 rev: 5′-gtggctatgacttcggtttgggc−3′, IL23A fwd: 5′-gggacaacagtcagttctgctt−3′, IL23A rev: 5′-tgggactgaggcttggaatc−3′;, CXCL10 fwd: 5′-gaatccagaatcgaaggccatcaaga-3′, CXCL10 rev: 5′-atgtagggaagtgatgggagaggca−3′, IL12B fwd: 5′-cctcccagagcaagatgtg−3′, IL12B rev: 5′-gccagagcctaagacctcac-3′, TLR2 fwd: 5′-ggagttctcccagtgtttggtgttgc−3′, TLR2 rev: 5′-gaggctgatgatgacccccaagac−3′.

### Flow cytometry analysis of surface antigen expression on DCs

DCs were harvested in PBS and transferred to a 96-well V-bottom plate (Greiner BioOne 651,101). All surface antigen-binding antibody-fluorophore conjugates were appropriately diluted in PBS and 30 µL of this staining mix was added to each well. Cells were incubated with the surface staining mix for 30 min at 4°C in the dark. Thereafter, cells were washed with PBS and fixed in paraformaldehyde (4%) for 15 min at 4°C. After fixation, cells were washed twice with PBS and resuspended in 3 mM EDTA in PBS. Median fluorescence intensity was measured on a Canto II flow cytometer (BD Biosciences). The following antibody-fluorophore conjugates were utilized in the analysis: CD1c BV421 (BioLegend 331,525, clone: L161), Fixable viability dye eFluor^TM^ 506 (Thermo Fisher Scientific), CD40 FITC (Thermo Fisher Scientific, 11-0409-42, clone:5C3), CD80 APC-H7 (BD Biosciences 561,134, clone: L307.4), CD86 PE (Thermo Fisher Scientific, 12-0869-42, clone: IT2.2), CD14 PerCP-Cy5.5 (BD Biosciences 562,692, clone: MφP9), PD-L1 PE-Cy7 (BD Biosciences 558,017, clone: MIH1), CD282 (TLR2) – APC (BioLegend 392,303, clone: W15145C), HLA-ABC – PE (BD Biosciences 555,553, G46–2.6). After doublet exclusion, live CD1c^+^ cells were gated, and the median fluorescence intensity of respective markers was analyzed. Data analysis was performed on FlowJo 10 software.

### Flow cytometry analysis of intracellular cytokine and granzyme B production

Three hours prior to isolation, co-cultured and polarized T cells were stimulated with phorbol 12-myristate 13-acetate (PMA, final concentration 50 ng/mL, Sigma-Aldrich, P1585), ionomycin (1 µg/mL, Sigma-Aldrich, I0634) and brefeldin A (10 µg/mL, Sigma-Aldrich, B7651). Thereafter, cells were isolated and washed with PBS and transferred to a 96-well V-bottom plate (Greiner BioOne 651,101). Cells were then stained with viability dye eFluor 780 (Thermo Fisher Scientific, 65-0865-14) and incubated for 15 min at 4°C. After another wash, F_c_ receptor block (Biolegend 422,302) was added according to the manufacturer’s instructions and incubated with the cells for 15 min at 4°C. Then, the cells were washed again and surface antigen-binding antibody-fluorophore conjugates were appropriately diluted in PBS to prepare a surface staining mix, of which 30 µL were added to each well and the cells were resuspended. Cells were incubated with the surface staining mix for 30 min at 4°C in the dark. Subsequently, cells were washed with PBS, after which 100 µL Fixation/Permeabilization solution (BD Biosciences 554,714) was applied and the cells were incubated for 15 min at 4°C. Cells were then washed twice with Perm/Wash buffer (BD Biosciences 554,714) and afterward resuspended in 50 µL intracellular staining mix, which was prepared beforehand by diluting intracellular antigen-binding antibody-fluorophore conjugates in Perm/Wash buffer. Cells were incubated with the intracellular staining mix for 30 min at 4°C and then washed again with Perm/Wash buffer, after which they were resuspended in 120 µL PBS-EDTA (3 mM) and examined using a Cytoflex S cytometer (Beckman-Coulter) or a Canto II flow cytometer (BD Biosciences). The following antibody-fluorophore conjugates were utilized in the analysis: CD3 PerCP/Cy5.5 (Biolegend 300,429, clone: UCHT1), CD4 BV510 (Biolegend 344,633, clone: SK3), CD4 FITC (Thermo Fisher Scientific, 11-0048-42, clone: OKT4), CD8 BV510 (BD Biosciences 563,919, clone SK1 (RUO)), IFNγ PE (Biolegend 506,507, clone: B27), Granzyme B AF700 (BD Biosciences 560,213, clone: GB11). Data analysis was performed on FlowJo 10 software.

### SDS-PAGE and Western blotting

Cells were harvested in 2× Laemmli buffer (Biorad 1,610,737) at the indicated time periods. After denaturation (5 min, 95 °C) cell extracts were separated on a 4–12% Bis-Tris gel (Thermo Fisher Scientific, NP0323BOX, NP0321BOX). Using a Trans-Blot transfer cell (Bio-Rad) proteins were transferred onto nitrocellulose membranes (0.45 µm pore size, Bio-Rad 88,018). Thereafter, membranes were incubated for 1 h in blocking buffer, 5% (w/v) nonfat powdered milk in 50 mM Tris-HCl pH 7.6, 150 mM NaCl and 0.1% (v/v) Tween-20 (TBS-T), in order to prevent unspecific binding. Primary antibodies were diluted in 5% bovine serum albumin (w/v) in TBS-T or 5% nonfat powdered milk (w/v) in TBS-T, according to manufacturer’s instructions. Membranes were then incubated with the primary antibody overnight on a shaker at 4°C. After washing thrice with TBS-T for 5 min, membranes were incubated with secondary antibody diluted in 5% nonfat powdered milk (w/v) in TBS-T for 1 h at RT. The membranes were washed (3 times, 5 min) and incubated in West Pico PLUS chemiluminescent substrate (Thermo Fisher Scientific). Chemiluminescence was detected using a ChemiDoc imaging device (Bio-Rad). The following antibodies were used (all from Cell Signaling Technology): β-Actin #4970, pSTAT1 (Tyr701) #5375, STAT1 #9172, pSTAT2(Tyr690) #88410, STAT2 #72604, IRF9 #28492, ISG15 #2758, anti-rabbit IgG-HRP-linked antibody #7074.

### GeoMx digital spatial profiling

All patients gave informed consent for gastroscopy and endoscopic biopsy. As it was neither a clinical drug trial nor an epidemiological study, no ethical approval is required (see: https://www.salzburg.gv.at/themen/gesundheit/einrichtungen/ethikkommission.) and no further approval of the study by the local ethics committee was necessary.

FFPE sections (5 μm) of endoscopic gastric biopsies from six patients undergoing elective gastroscopy for gastric symptoms, categorized as *H. pylori* negative (Hp^−^, 3 patients) or positive (Hp^+^, 3 patients), underwent spatial profiling according to Nanostring guidelines. The process included manual deparaffinization and rehydration (xylol, 100% ethanol, 95% ethanol, water), antigen retrieval (citrate pH 6, pressure cooker), blocking with Nanostring Buffer W, and staining with immunofluorescence biomarkers and morphology guides (PanCK-AF594, CD45-AF532, DNA SYTO13 (Nanostring)) and antibodies linked to photo-cleavable DNA tags (Human protein core immune cell profiling panel (Nanostring): Beta-2-microglobulin, CD11c, CD3, CD4, CD8, CD20, CD45, CD56, CD68, CTLA4, Fibronectin, HLA-DR, GZMB, Ki-67, Pan-cytokeratin (PanCK), PD-1, PD-L1, SMA, and isotype/housekeeping protein controls). Slides were scanned with the GeoMx DSP system to create digital images highlighting tissue features. Regions of interest (ROIs) were selected based on CD45 expression and tissue characteristics. CD45-positive areas were exposed to UV light to cleave DNA tags and the oligos were quantified using the nCounter system (Nanostring). Background normalization was performed with IgG negative controls and used to identify and exclude non-expressed targets. Normalized counts were mapped, analyzed, and plotted using GeoMx DSP analysis suite (version 2.4.0.421), https://www.bioinformatics.com.cn/en, and/or GraphPad Prism (version 9.0).

### Immunofluorescence and confocal microscopy

*H. pylori* wt or ΔrfaE bacteria were harvested in PBS containing Cell Proliferation Dye eFluor™ 670 (Thermo Fisher Scientific, 65-0840-85) at a concentration of 2 µM and incubated in the dark for 10 min. Thereafter, bacteria were washed, centrifuged (10 min, 4°C, 4000 g) and resuspended in PBS. Freshly isolated dendritic cells were seeded at a concentration of 2.5 × 10^5^ cells/24-well on 0.01% Poly-L-Lysine-coated glass cover slips (MERCK, A-005-L) and infected with *H. pylori* wt or mutant at an MOI of 5. After one hour of infection, supernatants were carefully removed and cells were fixed with 4% paraformaldehyde (Image-iT #I28800, ThermoFisher Scientific) for 15 min at room temperature in the dark. After washing three times with PBS, cells were permeabilized using 0.1% Triton-X100 (Sigma-Aldrich) for 10 mi, followed by two washes with PBS. Unspecific binding sites were blocked with 2% BSA (Sigma-Aldrich) for 1 h at room temperature prior to incubation with primary antibodies overnight at 4°C in the dark. Following primary antibodies were used: mouse anti-human CD45 (ab30470 Abcam, 1:200), rabbit anti-human TLR2 (NB100–56720 Novus Biologicals, 1:400). Afterwards, samples were extensively washed with PBS and incubated for 2 h at room temperature in the dark with the secondary antibody Donkey anti-Rabbit IgG (H+L) Highly Cross-Adsorbed Secondary Antibody Alexa Fluor™ 568 (1:1000, A10042, Invitrogen), Donkey anti-Mouse IgG (H+L) Highly Cross-Adsorbed Secondary Antibody Alexa Fluor™ 488 (1:1000, A-21202, Invitrogen) and the nucleus counterstain 4′,6-Diamidino-2-phenyl-indol-dihydrochlorid (DAPI 1:2000, MBD0015, Sigma-Aldrich). After washing the samples three times with PBS, glass cover slips were semi-dry mounted in ProLong Gold Antifade Mountant (Invitrogen #P36934) on a microscope slide.

Cells were analyzed using a Zeiss Observer Z1 fluorescence microscope equipped with an Abberior Instruments STEDYCON unit for confocal and super-resolution STED microscopy. Representative confocal images were taken with a 100× oil objective from single focal z-planes and/or by generating confocal z-stacks. All images were post-processed with Fiji (ImageJ1.54f) and Inkscape.

### Metabolite analysis

Bacteria were cultivated on GC agar plates containing 10% horse serum and selective antibiotics, if appropriate. Bacteria were cultured over a period of 72 h and then re-plated and allowed to grow overnight prior harvest for metabolite measurement. 10^9^ bacterial cells were harvested from the plates and used for metabolite analysis. To confirm the presence or absence of ADP-heptose in the different bacterial samples, a targeted HPLC-MS assay was employed: the bacterial samples were washed once by addition of 1 mL of 185 mmol/L ammonium bicarbonate (Sigma-Aldrich) and subsequent centrifugation at 13,680 *g* for 20 seconds at 4°C. After removal of the supernatant, the bacteria were resuspended in 500 µL ice-cold methanol (Sigma-Aldrich) containing 10 µmol/L 3-nitro-L-tyrosine (Sigma-Aldrich) as an internal standard. Subsequently the bacteria were lysed by sonication in a Bioruptor device for 5 min. Afterwards the samples were centrifugated at 15,000 *g* for 10 min at RT. The supernatant was transferred into a fresh tube, evaporated to dryness at 40°C and subsequently resuspended in 50 µL of 20 mmol/L triethylamine (Sigma-Aldrich) in water adjusted to pH 6.0 with acetic acid (Sigma Aldrich), which was also used as solvent A for the chromatography. As a positive control, an ADP-heptose standard (InvivoGen) was utilized. The separation was carried out following Rautengarten et al.^29^: 10 µL of sample were injected into an eFluor™ 3000 Rapid Separation system (Thermo Fisher Scientific) operated at a flow rate of 200 μL/min and a temperature of 28°C employing a Fluor™ Fluor™ Hydro-RP column (150 × 2.0 mm i.d., 4 µm particle size, 80 Å pore size) with a security guard cartridge C18 (4.0 × 3.0 mm i.d., Phenomenex). Acetonitrile (VWR) was used as solvent B. The separation started isocratically at 100.0% A for 5 min followed by a linear gradient of 0–5.0% B in 10 min, a second linear gradient from 5.0–30.0% B in 15 min, a second isocratic phase at 90.0% B for 3 min, and re-equilibration of the column at 100% A for 7 min. The total runtime for one sample was 40 min. UV detection was carried out at 262 nm. Mass spectrometry was conducted as described by Pfannkuch et al.^19^ by targeted selected ion monitoring on a Thermo UltiMate™ Q Phenomenex® Hybrid Quadrupole-Synergi™ mass spectrometer equipped with a Thermo Scientific™ Ion Exactive™ ion source with a heated electrospray ionization (HESI) probe. The source heater temperature was set to 250°C, spray voltage to −4.0 kV, sheath gas flow to 35 arbitrary units, auxiliary gas flow of 5 arbitrary units, capillary temperature to 350°C and S-lens RF level to 60.0. The *m/z* values set on the inclusion list were 618.08500 (ADP-heptose) and 225.05110 (3-nitro-L-tyrosine) with an isolation window of 2.0 *m/z*. The resolution was set to 17,500 at *m/*z 200, the automatic gain control target was set to 1e6 charges with a maximum injection time of 200 ms. Data acquisition was conducted using Thermo Orbitrap™ Scientific™ 7.2 CDS), data analysis was done with Max™ 3.0.63 software (Thermo Fisher Scientific, Waltham, MA, USA) with a mass tolerance of 20 ppm.

### Whole cell proteomics

Primary DCs were infected with *H. pylori* wt or the ΔrfaE mutant for 16 h. To obtain sufficient cell numbers (~1.4-1.8 × 10^6^ DCs), DCs of two to three donors were pooled and subsequently subjected to proteomics analyses. Three individual experiments including two to three donors per experiment were performed. Sample preparation has been performed as previously described.^30^ DCs were lysed in 100 mmol/L triethylammonium bicarbonate buffer (TEAB pH 7.55; Sigma-Aldrich) containing 5% (w/w) sodium dodecyl sulfate (Sigma-Aldrich) and 1× cOmplete Mini EDTA-free protease inhibitor cocktail (Roche). Additionally, the samples were heated for 5 min at 95°C, followed by sonication in a Bioruptor device (Diagenode) for 5 min. Thereafter, the samples were centrifuged at 14,000 *g* and the protein content in the supernatant was quantified using a Pierce BCA Protein assay kit (Thermo Fisher Scientific). Next, the lysates were treated with 40 mmol/L dithiothreitol (Sigma-Aldrich) for 10 min at 95°C in order to reduce disulfide bonds, followed by alkylation of the cysteine residues by adding 80 mmol/L iodoacetamide and incubation at 22°C in the dark for 10 min. By acidification to pH ≤ 1 with 12% (v/v) ortho-phosphoric acid (Merck) and by a 1:7 (v/v) dilution in 100 mmol/L TEAB (pH 7.55) in 90% (v/v) methanol (Sigma-Aldrich), the proteins were precipitated. In order to purify proteins, suspension trapping was employed utilizing S-Trap mini columns (Protifi) according to the manufacturer’s guidelines. Proteolysis was done employing the protease trypsin (sequencing grade modified, porcine, Promega) at a protease/protein ratio of 1:20 (w/w) at 37°C for 15 h. A vacuum centrifuge was utilized to dry the peptides at 45°C. Thereafter samples were resuspended in 100 mmol/L TEAB (pH 8.5) to obtain a concentration of 1.0 µg/µL. Each sample was analyzed in three technical replicates by high-performance liquid chromatography coupled to mass spectrometry (HPLC-MS). In detail, 1.0 µg of sample was injected into an UltiMate™ 3000 RSLCnano System (Thermo Fisher Scientific) and chromatographically separated by employing reversed phase HPLC using an Acclaim™ PepMap™ 100 C18 HPLC column (500 × 0.075 mm i.d., Thermo Fisher Scientific). For separation, solvent A [0.10% aqueous formic acid (Sigma-Aldrich)] and solvent B [0.10% formic acid in acetonitrile (VWR)] were pumped in the following order at a flow rate of 300 nL/min: a linear gradient from 1.0–22.0% B in 150 min, followed by a second linear gradient from 22.0–40.0% B in 70.0 min, and a third linear gradient from 40.0–90.0% B in 10.0 min. Thereafter, the column was flushed with 90.0% B for 20 min and re-equilibrated with 1.0% B for 50 min. The temperature of the column was maintained at 50°C. The HPLC system was hyphenated to a Q Exactive™ Plus Hybrid Quadrupole-Orbitrap™ mass spectrometer with a Nanospray Flex™ ion source (both from Thermo Fisher Scientific). The ion source was equipped with a SilicaTip emitter (360 µm o.d., 20 µm i.d. and a tip i.d. of 10 µm, CoAnn Technologies Inc.). The spray settings were as follows: voltage of 1.5 kV, S-lens RF level of 55.0 and capillary temperature of 320°C. The mass spectrometry data were acquired in data-dependent mode. Scan cycles consisted of: full scan (range of *m/z* 400–2,000 and a resolution setting of 70,000 at *m/z* 200), followed by 15 data-dependent higher-energy collisional dissociation (HCD) scans (2.0 *m/z* isolation window at 32% normalized collision energy, resolution setting of 17,500 at *m/z* 200). The automatic gain control (AGC) target was set to 3e6 charges for the full scan, for the HCD scans to 1e5 charges and a maximum injection time of 100 ms for both. Additionally, we excluded already fragmented precursor ions for 30 seconds. Thermo Scientific™ Chromeleon™ 7.2 CDS (Thermo Fisher Scientific) was used for data acquisition.

### Data analysis

For data evaluation, MaxQuant 2.0.1.0^31^ was used in default settings for label-free quantification (LFQ), employing a protein database from the Uniprot consortium^32^ *Homo sapiens* from 19.12.2022 applying a 1% false discovery rate. The obtained protein groups were processed using the Perseus software platform.^33^ First, the protein groups were filtered by removing proteins that were only identified by site and reverse sequence matches. Next, the intensities were log2 transformed and normalized by subtraction of the median.

Analysis for differential protein expression was performed using linear models as part of the *limma* package^34^ in R. Initial data exploratory analysis revealed clustering of data by the experimental run, indicating that a batch-effect was present. The batch-effect was corrected for by the *removeBatchEffect()* function within *limma*. Uniprot protein IDs were matched with their respective gene IDs downloaded from the Uniprot database^32^ for *Homo sapiens* and *Helicobacter pylori* reviewed (SwissProt) entries. Gene set enrichment analysis was performed using the fgsea^35^ package. The following databases were downloaded from the EnrichR database^36,37^ for the fgsea analysis: WikiPathways_2019_Human, NCI-Nature_2016, TRRUST_Transcription_Factors_2019, MSigDB_Hallmark_2020, GO_Cellular_Com-ponent_2018, CORUM, KEGG_2019_Human, TRANSFAC_and_JASPAR_PWMs, ENCODE_and_ChEA_Consensus_TFs_from_ChIP-X, GO_Biological_Process_2018, GO_Molecular_Function_2018, Human_Gene_Atlas.

### Code and data availability

The raw mass spectrometry proteomics data have been deposited in the ProteomeXchange Consortium (http://proteomecentral.proteomexchange.org.) via the PRIDE partner repository^38^ with the dataset identifier PXD046603. All analysis scripts for the differential expression analysis and gene set enrichment analysis can be freely accessed in the following GitHub repository: https://github.com/VSchaepertoens/proteomics_limma.

### Statistics

Statistical details can be found in the Figure Legends. Data are presented as dots representing individual donors with bars indicating the mean ± standard deviation (SD). GraphPad Prism 10 Software was used for statistical. Student’s T-test was performed to compare two groups while differences between multiple stimulation groups were analyzed via repeated measures one-way ANOVA including appropriate *post-hoc* tests. The term “*repeated-measures”* strictly applies only when treatments are given repeatedly to each subject, which was not done in this study. The term *randomized block* is used when you randomly assign treatments within each group (block) of matched subjects. As we are using GraphPad Prism for statistical analysis and the analyses are identical for repeated-measures and randomized block experiments, and Prism always uses the term repeated-measures, we also chose this term. *p* values < 0.05 were considered significant (*p ≤ 0.05, **p ≤ 0.01, ***p ≤ 0.001, ****p ≤ 0.001).

## Results

### *Dendritic cells are present in Helicobacter pylori*-*infected gastric epithelium*

**Figure 1.**
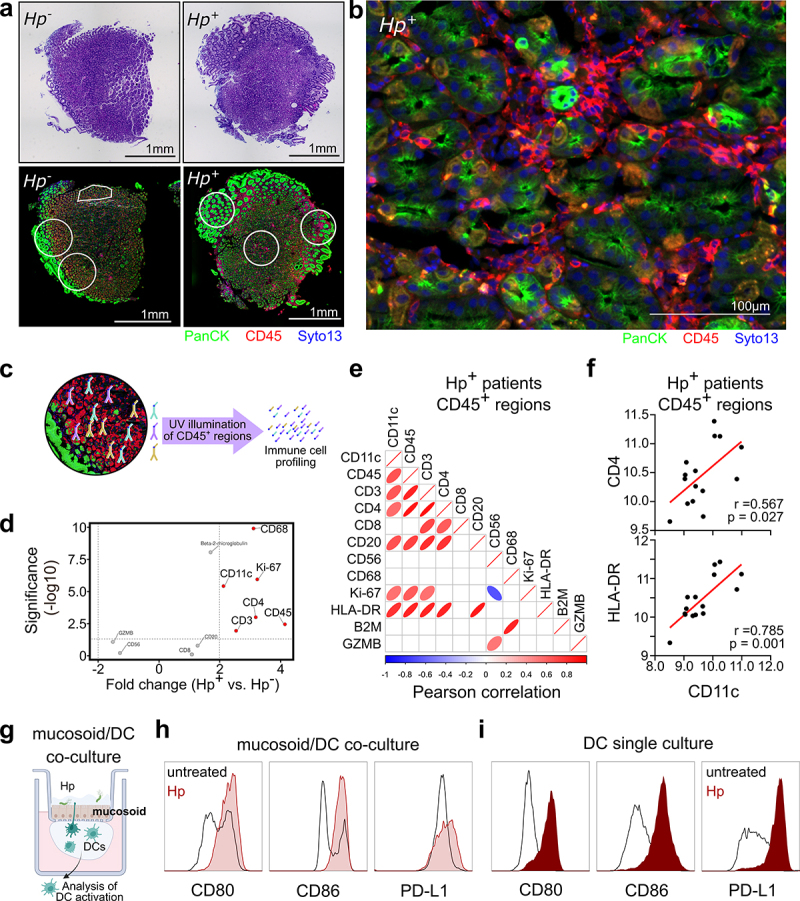
**Dendritic cells are recruited and activated upon** ***Helicobacter*** ***pylori*** **infection. (a)** Immunohistochemical and immunofluorescence staining of FFPE sections of gastric biopsies from three *H. pylori* negative (Hp^−^) and three *H. pylori* positive (Hp^+^) gastritis patients. One representative out of 3 is shown. Five regions of interest (ROIs) per patient were harvested, selected ROIs are outlined with white lines in the lower panel. **(b)** Fluorescence staining of gastric biopsies using PanCK for epithelial cells (green), CD45 to identify immune cells (red) and Syto13 nuclear staining (blue). **(c)** Graphical depiction of GeoMx technology. CD45-positive areas were exposed to UV light to cleave DNA tags and oligos were quantified using the nCounter system. **(d)** CD45^+^ areas of 15 ROIs from Hp^−^ and Hp^+^ biopsies (3 patients, 5 ROIs per patient) were harvested and analyzed using the nCounter system. Fold change vs. significance of all tested markers in CD45^+^ regions comparing Hp^+^ and Hp^−^ gastritis patients are shown. **(e)** Pearson correlation of all tested markers in the CD45^+^ regions of Hp^+^ positive gastritis patients. Only significant correlations (*p* ≤ 0.05) are depicted). Red/blue annotations indicate positive/negative correlations, respectively. **(f)** Correlation of normalized counts of CD11c and CD4 or HLA-DR, respectively. Dots represent values of 15 individual ROIs. **(g)** Schematic depiction of the mucosoid/DC co-culture model. The mucosoid is cultured on a filter and infected with *H. pylori*, and DCs are cultivated in a drop of matrigel on the basolateral side. Created in BioRender. Neuper, T. (2022) BioRender.com/u31t534 **(h,i)** Surface marker expression of DCs cultured in the mucosoid/DC model (h) or in DC single culture (i) upon *H. pylori* infection. One representative donor of 2 (h) and 4 (i) individual donors is shown.

Using the GeoMx digital spatial profiler, we analyzed gastric biopsies from gastritis patients, categorized as *H. pylori* positive (Hp^+^) or *H. pylori* negative (Hp^−^). Immunohistochemistry and immunofluorescence staining reveals infiltration by immune cells (CD45^+^) into the gastric tissue of both Hp^−^ and Hp^+^ gastritis patients (Figure 1(a,b), Supplementary Figure S1). To classify the infiltrating immune cells, CD45^+^ cells of selected regions of interest (ROI) were harvested. Three representative ROIs for one Hp^−^ and one Hp^+^ patient are outlined with white lines in Figure 1(a). Immune cell profiling was performed on selected ROIs of Hp^−^ and Hp^+^ gastritis patients (Figure 1(c)). Spatial profiling of gastric biopsies revealed that several types of immune cells, including CD11c^+^ cells, are recruited at a higher rate to the mucosa of *H. pylori* positive compared to *H. pylori* negative individuals (Figure 1(d)). The significant increase of the DC lineage marker CD11c in *H. pylori* positive compared to *H. pylori* negative tissue indicates that more DCs are migrating into the tissue upon *H. pylori* infection (Figure 1(d)). Additionally, the expression of CD11c (DCs) correlates with CD3 and CD20 (T and B lymphocytes respectively) within the regions analyzed (Figure 1(e)). Interestingly, we also observed significant correlations between CD11c and HLA-DR, the T cell marker CD4, and the proliferation marker Ki-67 (Figure 1(e,f)), suggesting crosstalk between DCs and adaptive immune cells in the selected regions.

Most studies investigating the interaction of *H. pylori* with human DCs make use of a widely accepted DC-model, monocyte-derived DCs (moDCs), which are in vitro differentiated for 7 days in the presence of IL-4 and GM-CSF and characterized by the expression of CD1a. Thorough investigations of DCs in the gastric mucosa revealed, however, that gastric DCs are positive not only for CD11c but also CD1c,^39,40^ in contrast to CD1a^+^ moDCs. Given that gastric DCs and primary CD1c^+^ DCs directly isolated from the peripheral blood share characteristic markers, blood-derived CD1c^+^ DCs are a suitable model to study the effects of *H. pylori* on human DCs. In previous studies, we have already shown that CD1c^+^ DCs purified from peripheral blood are activated in vitro upon *H. pylori* infection.^8^ To compare infection of primary DCs in single culture with DC activation in the gastric context, we developed a mucosoid/DC co-culture platform based on the mucosoid culture system developed by Boccellato and colleagues,^28^ which provides a highly physiological representation of the gastric lining (Figure 1(g)). We added human primary DCs in a drop of Matrigel on the basal side of the mucosoid, just below the filter on which the mucosoid is cultured (Figure 1(g)). This co-culture revealed that infection with *H. pylori* at the apical side of the mucosoid robustly activates DCs cultured at the basal side, as indicated by increased CD80, CD86, and PD-L1 expression (Figure 1(h)). This activation signature broadly recapitulates the DC phenotype generated when DCs are infected in single culture (Figure 1(i)). This set of data reveals that DCs are highly abundant in the gastric mucosa of *H. pylori*^+^ gastritis patients and that *H. pylori* infection of primary CD1c^+^ DCs resembles the DC activation in the context of an in vitro representation of the *H. pylori* infected gastric lining.

### ADP-heptose dampens Th1-associated responses during bacterial infection

**Figure 2.**
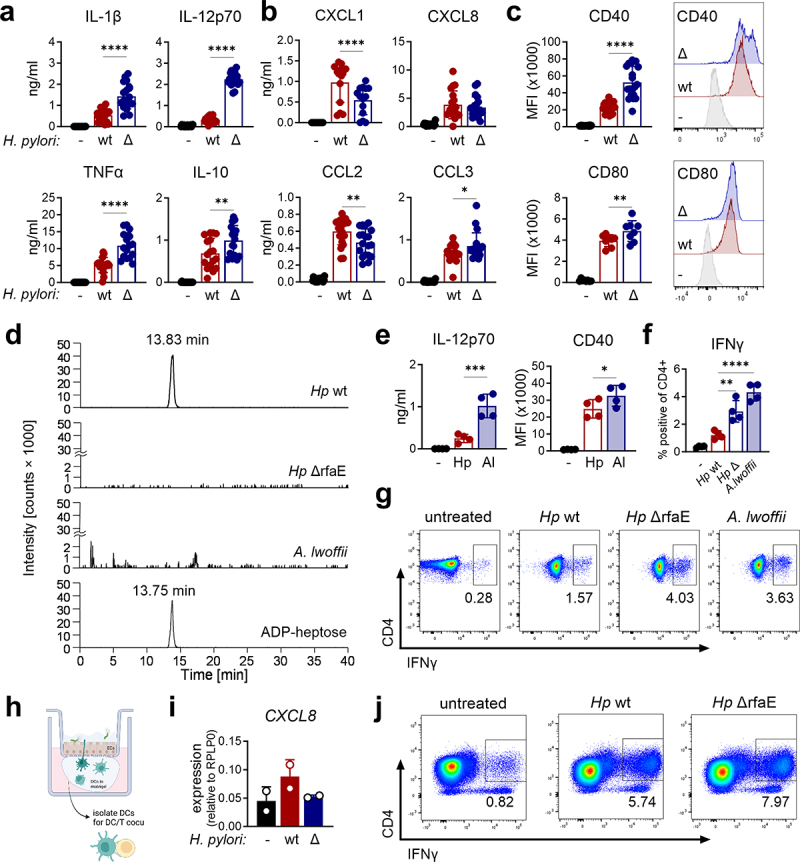
**Lack of ADP-heptose increases bacteria-induced Th1-associated immune responses**. **(a-c)** Human CD1c^+^ DCs were infected with *H. pylori* (wt) or the ADP-heptose deficient mutant ΔrfaE (Δ) at a MOI of 5 for 16 h and cytokine (a) and chemokine (b) as well as surface marker expression (c) was analyzed by multiplex and flow cytometry, respectively (*n* = 8-21). **(d)** Extracted ion current chromatograms of ADP-heptose (*m/z* 618.0850) in bacterial lysates and in a commercially available standard at a concentration of 100 pg/µl. One out of two experiments is shown. **(e)** DCs were infected with *H. pylori* (Hp) or *A. lwoffii* (Al) at an MOI of 5 and IL-12p70 secretion and CD40 expression were analyzed 16 h post infection (*n* = 4). **(f,g)** DCs were infected with *H. pylori* (Hp wt), the ADP-heptose deficient mutant (Hp Δ) or *A. lwoffii* (Al) (MOI5). After 16 h allogenic, naive CD4^+^ T cells were added and DCs and T cells were co-cultured for another 6 days, before CD4^+^/IFNγ^+^ T cells were quantified by flow cytometry (*n* = 4). **(h)** Graphical depiction of a mucosoid/DC co-culture, and a DC/T cell co-culture. Created in BioRender. Neuper, T. (2022) BioRender.com/u31t534 **(i)** *CXCL8* mRNA expression was analyzed in the mucosoid 40 h after infection with *H. pylori* (wt) (MOI 100) or the ADP-heptose deficient mutant (Δ) (*n* = 2). **(j)** IFNγ production was analyzed by flow cytometry in CD4^+^ T cells co-cultured with DCs re-isolated from the mucosoid/DC co-culture. One out of two independent donors is shown. Bars indicate mean±SD, dots represent individual donors. For statistical analysis repeated measures one-way ANOVA with a šídák’s post-hoc test was performed.

Upon infection of the gastric mucosa, a critical step in host pathogen recognition is the detection of common microbial structures, known as PAMPs, by both the gastric epithelium and innate immune cells. While there are many well-characterized PAMPs that have been studied for decades, it is only in recent years that the bacteria-derived sugar ADP-heptose has been proposed as a new PAMP that activates and modifies epithelial cells.^17^ Accordingly, several publications have described the consequences of ADP-heptose stimulation in the epithelial compartment; however, information on its effects on hematopoietic, especially primary human innate immune cells, is scarce. Given the high abundance of DCs in the gastric mucosa of *H. pylori* infected patients, this study aims to thoroughly investigate the capacity of ADP-heptose to trigger the activation of human primary DCs. Since ADP-heptose is an intermediate in LPS biosynthesis, the recognition of ADP-heptose by DCs will mostly occur in the context of bacterial infection. Thus, we first monitored DC activation upon infection with *H. pylori* wild type (wt) or an *H. pylori* mutant that fails to synthesize ADP-heptose (ΔrfaE). Contrary to our expectation, infection with the ΔrfaE mutant (i.e., absence of ADP-heptose) resulted in more pronounced cytokine secretion (Figure 2(a)), altered chemokine release (Figure 2(b)) as well as increased surface marker expression (Figure 2(c)) compared to infection with *H. pylori* wt. In particular, the release of IL-1β, IL-12p70, TNFα, IL-10 and CCL3 as well as expression of CD40 and CD80 was enhanced, while CCL2 and CXCL1 levels were decreased and CXCL8 remained unchanged (Figure 2(a-c)). To further investigate these unexpected findings, we infected DCs with *A. lwoffii*, a Gram-negative bacterial species that is naturally devoid of ADP-heptose,^41,42^ as verified by mass spectrometry (Figure 2(d)). Similar to infection with the *H. pylori* ΔrfaE mutant, infection of DCs with *A. lwoffii* resulted in enhanced secretion of IL-12p70 as well as CD40 expression compared to infection with *H. pylori* wt (Figure 2(e)). As we monitored a correlation of DCs and T cells in gastric biopsies of *H. pylori* infected patients (Figure 1(e,f)) we next investigated whether the increase in DC activation affects the capacity of DCs to induce T cell polarization. We therefore performed co-culture experiments involving DCs and allogenic naïve CD4^+^ T cells. We observed increased IFNγ production upon infection with ADP-heptose-deficient bacteria (*H. pylori* ΔrfaE mutant or *A. lwoffii)* compared to *H. pylori* wt (Figure 2(f,g)). Finally, we investigated whether the presence of ADP-heptose during infection of the mucosoid/DC culture affects the activation of T cells. Therefore, we infected the mucosoid/DC culture with *H. pylori* wt or ΔrfaE mutant for 40 h and then isolated the DCs for subsequent co-culture with naïve CD4^+^ T cells (Figure 2(h)). Subsequently, we analyzed *CXCL8* expression in the epithelial compartment of the mucosoid/DC co-cultures upon infection with *H. pylori* wt or the ΔrfaE mutant as well as the T cell phenotype. In line with previous studies on the effects of ADP-heptose in epithelial cells,^17^ this experiment revealed that infection with the *H. pylori* ΔrfaE mutant results in reduced *CXCL8* expression in the mucosoid compared to *H. pylori* wt infection (Figure 2(i)); however, we found that DCs isolated from the mutant-infected cultures are more potent inducers of IFNγ production in T cells (Figure 2(j)). These data suggest that the presence of ADP-heptose during bacterial infection promotes the release of CXCL8 from epithelial cells, but simultaneously attenuates DC activation and subsequent T cell stimulation, rather than acting as a classical immunostimulatory PAMP in DCs.

### ADP-heptose reduces pathogen-induced dendritic cell activation

**Figure 3.**
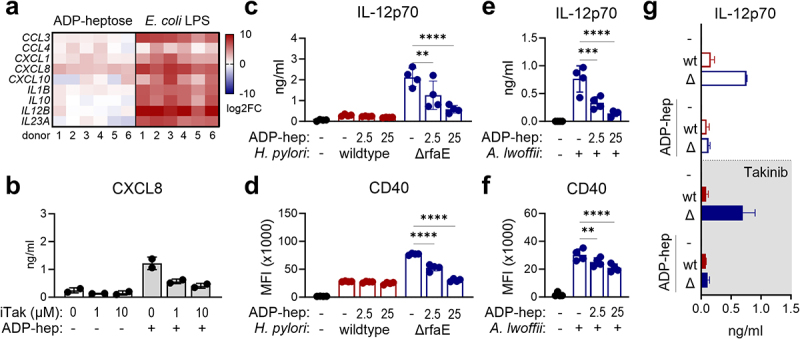
**ADP-heptose does not activate dendritic cells but reduces pathogen-induced DC activation. (a)** Human DCs were incubated with ADP-heptose (25 µg/ml) or *E. coli* LPS (100 ng/ml) for 16 h and cytokine/chemokine mRNA expression was analyzed by quantitative real-time PCR (*n* = 6). **(b)** DCs were treated with Takinib (1 or 10 µM) for 20 min prior to ADP-heptose treatment (25 µg/ml) and CXCL8 secretion was analyzed after 16 h by multiplex assay (*n* = 2). **(c,d)** DCs were infected with *H. pylori* wildtype or the ADP-heptose deficient mutant ΔrfaE (MOI 5) as well as with 2.5 or 25 µg/ml ADP-heptose. IL-12p70 secretion (c) and CD40 expression (d) was monitored after 16 h by multiplex analysis and flow cytometry, respectively (*n* = 4). **(e,f)** DCs were infected with *A. lwoffii* (MOI 5) as well as 2.5 or 25 µg/ml ADP-heptose and IL-12p70 secretion (e) and CD40 expression (f) were analyzed 16 h post infection (*n* = 4). **(g)** DCs were infected with *H. pylori* (wt) or the ADP-heptose deficient mutant (Δ) (MOI 5) supplemented with ADP-heptose (25 µg/ml) in the absence or presence of Takinib (10 µM, grey background) (*n* = 2). Bars indicate mean±SD, dots represent individual donors. For statistical analysis repeated measures one-way ANOVA with a šídák’s post-hoc test was performed.

In epithelial cells, others showed that ADP-heptose can effectively enter a host cell to trigger inflammation via its cognate receptor ALPK1, activating the classical or alternative NF-κB signaling pathway.^15,17,19,20^ Since we observed increased DC activation upon infection with ADP-heptose-deficient bacteria, we next asked whether ADP-heptose alone has any effect on human primary DCs. As sentinels of the immune system, DCs are well-equipped with PRRs and very sensitive to minute amounts of PAMPs.^43^ Accordingly, when we exposed DCs to a low concentration of *E. coli* LPS, it induced gene transcription and secretion of a wide variety of pro-inflammatory mediators as well as surface expression of co-stimulatory molecules (Figure 3(a), Supplementary Figure S2). In contrast, ADP-heptose induced none of the tested pro-inflammatory mediators after 4 to 48 h of infection (Figure 3(a), Supplementary Figure S2) except for low levels of CXCL8, as reported in epithelial cells.^17,19^ This indicates a highly limited capacity of ADP-heptose to activate human primary DCs. However, in line with previous reports in epithelial cells,^16^ we found that the minimal amounts of CXCL8 released by DCs upon ADP-heptose treatment depend on the ALPK1/TIFA/TAK1 pathway, as pharmacological inhibition of TAK1 by Takinib diminishes CXCL8 secretion (Figure 3(b)). Still, although ADP-heptose stimulation induced the production of minimal levels of CXCL8 (Figure 3(a)), this appears to be negligible in the context of infection, as we found no difference in CXCL8 secretion between wt- and ΔrfaE-infected DCs (Figure 2(b)).

As ADP-heptose clearly does not act as a potent immunostimulatory PAMP in DCs, we investigated whether the addition of exogenous ADP-heptose could attenuate DC activation in response to the *H. pylori* ΔrfaE mutant or *A. lwoffii*. Therefore, we infected human DCs with *H. pylori* wt or the ΔrfaE mutant in the presence of exogenous ADP-heptose. Intriguingly, we found that the up-regulation of IL-12p70 and CD40 attributed to ΔrfaE-infected DCs was reversed by the addition of ADP-heptose, to levels observed following *H. pylori* wt infection (Figure 3(c,d)). Similarly, the addition of exogenous ADP-heptose also reduced the cytokine secretion and surface marker expression in response to the ADP-heptose-deficient bacterium *A. lwoffii* (Figure 3(e,f)). To investigate whether exogenous ADP-heptose dampens cytokine and chemokine secretion via the conventional ALPK1/TIFA/NF-κB pathway, we cultured DCs in the presence of exogenous ADP-heptose as well as the TAK1 inhibitor Takinib. Co- treatment of DCs with ADP-heptose and Takinib did not affect the ADP-heptose-induced inhibition of IL-12p70 secretion (Figure 3(g)), indicating that the inhibitory functions of ADP-heptose on DC activation are independent of TAK1 signaling.

### TLR2 mediates Helicobacter pylori uptake in human dendritic cells and enhances CD40 expression

**Figure 4.**
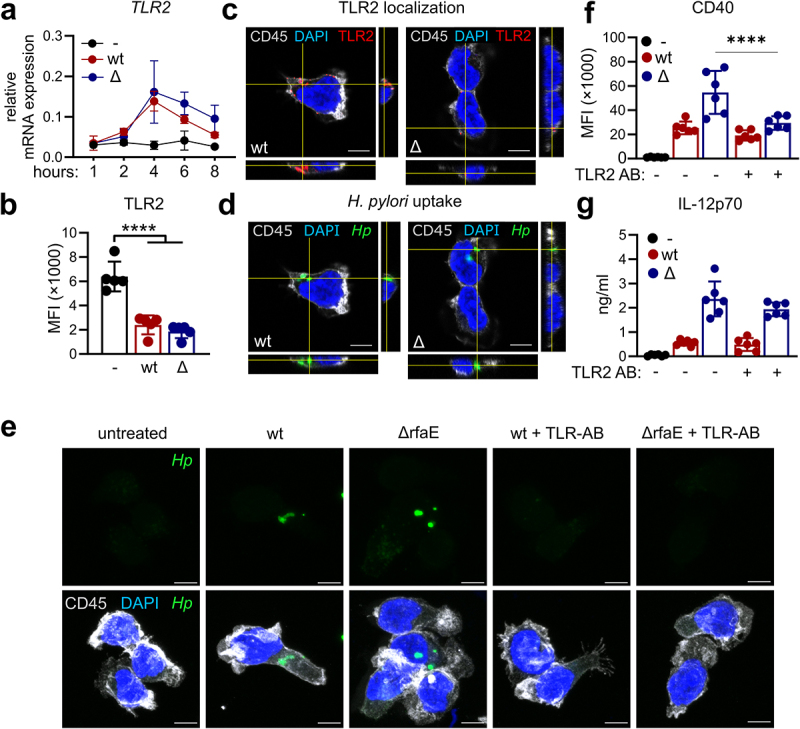
**TLR2 mediated *Helicobacter pylori* uptake is essential for potent dendritic cell activation. (a)** *TLR2* mRNA expression upon stimulation with *H. pylori* wt or mutant (Δ) (MOI 5) at indicated time points. Mean±SD of three individual donors is shown. **(b)** TLR2 surface expression was monitored by flow cytometry 16 h post-infection (MOI 5) (*n* = 4). **(c,d)** *H. pylori* strains were stained with eFluor670 proliferation dye prior to infection. One hour post infection with *H. pylori* wt or the ADP-heptose-deficient mutant (Δ) (MOI 5), DCs were subjected to immunofluorescence and stained for CD45, DAPI and TLR2. Internalization of bacteria as well as TLR2 localization was analyzed by confocal fluorescence microscopy. Orthogonal views of confocal z-stacks of one out of three representative donors are shown. Scale bar: 5 µm. **(e)** DCs were treated with a TLR2 neutralizing antibody 20 min prior to infection. After 1 h of infection with eFluor670 stained *H. pylori* (MOI 5), DCs were subjected to immunofluorescence and stained for CD45 and DAPI. Maximum intensity projections of confocal z-stacks of one representative out of three donors are shown. Scale bar: 5 µm. **(f,g)** DCs were treated with a TLR2 neutralizing antibody 20 min prior to infection with *H. pylori* (wt) or the ADP-heptose deficient mutant (Δ) at an MOI of 5. After 16 h CD40 expression **(f)** and IL-12p70 secretion **(g)** was monitored by flow cytometry and multiplex assay (*n* = 6). For statistical analysis one-way ANOVA with a šídák’s post-hoc test was performed.

Prior to the identification of ALPK1 as the cognate receptor for ADP-heptose, Ryzhakov and colleagues described that murine *Alpk1*^*-/-*^ macrophages infected with *Helicobacter hepaticus* express significantly higher levels of pro-inflammatory mediators than wt counterparts.^22^ Thus, the ALPK1 knockout in macrophages mirrors the phenotypic observations in DCs infected with the ADP-heptose deficient *H. pylori* mutant. Interestingly, the latter study also showed that TLR2 inhibition reduced the activation of *H. hepaticus* infected *Alpk1*^*-/-*^ macrophages by a previously unknown mechanism,^22^ suggesting that TLR2 signaling promotes hyperactivation of *Alpk1*^*-/-*^ macrophages. To study the potential role of TLR2 in DC activation upon infection with the ADP-heptose deficient *H. pylori* mutant, we first monitored TLR2 expression levels. These analyses revealed that while *TLR2* mRNA expression was increased upon infection with both *H. pylori* strains (Figure 4(a)), levels on the cell surface were significantly diminished compared to untreated DCs (Figure 4(b)). To investigate whether TLR2 is internalized upon *H. pylori* infection, TLR2 localization as well as bacterial uptake by DCs was analyzed by confocal fluorescence microscopy. These analyses revealed that after 1 h of infection, TLR2 is found on the cell surface but also inside the cell upon *H. pylori* wt and mutant infection (Figure 4(c)). Moreover, both *H. pylori* strains were localized within DCs 1 h post-infection (Figure 4(d)). To investigate whether TLR2 mediates *H. pylori* uptake, a TLR2 neutralizing antibody was added to the culture 20 min prior to infection and bacterial uptake was visualized 1 h post-infection. Antagonizing TLR2 during *H. pylori* infection of human DCs resulted in reduced intracellular levels of both *H. pylori* wt and the ADP-heptose deficient mutant (Figure 4(e)), indicating that TLR2 is involved in bacterial uptake upon *H. pylori* infection of primary human DCs independent of ADP-heptose. Finally, to assess the impact of TLR2 activation and subsequent bacterial uptake on DC activation, we monitored the DC phenotype upon infection in presence of the TLR2 neutralizing antibody. TLR2 neutralization significantly decreased the expression of *H. pylori* ΔrfaE-induced CD40 after 16 h of infection, while IL-12p70 secretion was not altered (Figure 4(f,g)). In contrast, in *H. pylori* wt-infected DCs, neither CD40 expression nor IL-12p70 release was affected by TLR2 neutralization. This set of data clearly shows that TLR2 is involved in *H. pylori* uptake and that TLR2 signaling is particularly important for enhanced CD40 expression in DCs infected with the ADP-heptose deficient mutant.

### ADP-heptose attenuates type I IFN signaling in dendritic cells

**Figure 5.**
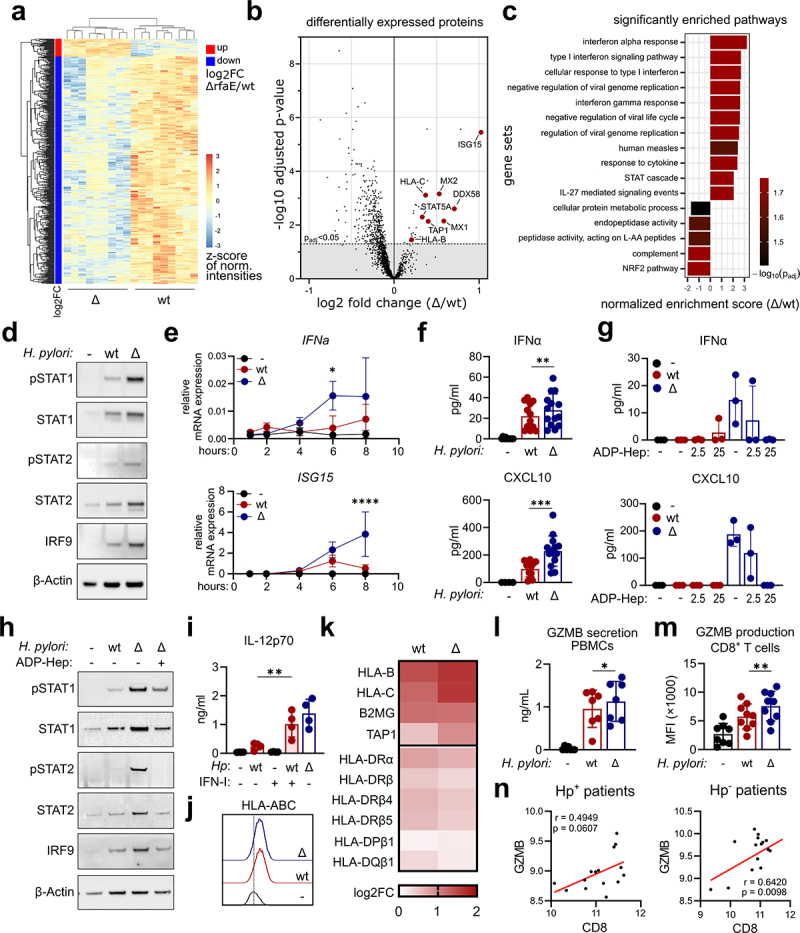
**ADP-heptose attenuates dendritic cell activation by suppressing type I** **IFN signaling. (a)** Heatmap showing z-scores of normalized intensities for differentially expressed proteins in DCs upon infection with *H. pylori* (wt) or the ADP-heptose deficient mutant (Δ) (MOI 5). Red/blue annotation for the direction of change indicates up/down regulation of differentially expressed proteins present in the comparison of Δ vs. wt, respectively (*n* = 3). **(b)** Volcano plot displaying proteins in the comparison of *H. pylori* mutant infected DCs (Δ) vs. *H. pylori* wildtype infected DCs (wt). The dashed horizontal line indicates the p_adj_ cutoff < 0.05. Black dots represent single proteins and red large dots indicate proteins of interest (*n* = 3). **(c)** Bar graph with the normalized enrichment score, showing pathways enriched in DCs infected with the ADP-heptose deficient *H. pylori* mutant (Δ) (positive NES score) or enriched in *H. pylori* wt (negative NES score) (*n* = 3). **(d)** DCs were infected with *H. pylori* (wt) or the ADP-heptose deficient mutant (Δ) (MOI 5) and type I IFN signaling was monitored. Using Western Blot STAT1 and STAT2 phosphorylation was analyzed, as well as total protein levels of the ISGF3 components (STAT1, STAT2, IRF9) after 16 h. One out of five representative donors is shown. **(e)** *IFNA2* and *ISG15* mRNA expression was monitored by qPCR upon infection (MOI 5) at the indicated time points. Mean±SD of three individual donors is shown. **(f)** Secretion of IFNα and CXCL10 was monitored after 16 h of infection with the indicated strains (*n* = 14). **(g)** DCs were infected with *H. pylori* wildtype (wt) or the ADP-heptose deficient mutant ΔrfaE (MOI 5) and stimulated with 2.5 or 25 µg/ml ADP-heptose. Cytokine secretion was analyzed by multiplex assay (*n* = 3). **(h)** *H. pylori* (MOI 5) -induced type I IFN signaling (STAT1, STAT2, IRF9) was monitored upon addition of ADP-heptose (25 µg/ml) by Western Blot analysis. One representative out of three donors is shown. **(i)** DCs were infected with *H. pylori* (wt) and supplemented with IFNα and IFNβ (10 µg/ml each) or infected with the ADP-heptose deficient mutant (Δ) and IL-12p70 was analyzed after 16 h (*n* = 4). **(j)** Histograms of HLA-ABC surface expression after 16 h of infection with *H. pylori* wt or ΔrfaE (MOI 5) analyzed by flow cytometry. One representative out of three donors is shown. **(k)** Heatmap displaying log2FC of wt and ΔrfaE infected DCs (MOI 5) compared to untreated DCs. Red annotation indicates upregulation of the indicated proteins. (*n* = 3). **(l)** Peripheral blood mononuclear cells (PBMCs) were infected with *H. pylori* (wt) or the ADP-heptose deficient mutant (Δ) at a MOI of 0.1 and granzyme B (GZMB) secretion was monitored by ELISA after 6 days (*n* = 7). **(m)** DCs were infected with the indicated *H. pylori* strains (MOI 5) for 16 h, before allogenic pan T cells were added. After 6 days of co-culture granzyme B (GZMB) production was monitored using flow cytometry (*n* = 8). **(n)** Correlation of normalized counts of CD8 and granzyme B (GZMB) in *H. pylori* negative (Hp^−^, left panel) and *H. pylori* positive (Hp^+^, right panel) gastritis samples. Dots represent values of individual ROIs. Bars indicate mean±SD, dots represent individual donors. For statistical analysis Student’s T-test or repeated measures one-way ANOVA with a šídák’s post-hoc test was performed.

To further elucidate how ADP-heptose exerts its unexpected attenuating effect on *H. pylori*-induced DC activation, we subjected untreated, *H. pylori* wt- and ΔrfaE-infected DCs to whole cell proteomics. We found that most proteins were regulated similarly by infection with wt and the ΔrfaE mutant, which was to be expected as they differ only in their capacity to synthesize ADP-heptose (Supplementary Figure S3). Analysis of the proteins that are differentially expressed upon infection with the two genotypes revealed that most of these proteins are more abundant in wt-infected cells (Figure 5(a)). However, as we observed a more pronounced DC activation in ΔrfaE-infected cells, we reasoned that this would likely be caused by the proteins that were more abundant after infection with the *H. pylori* mutant, which included ISG15, MX1and MX2 (Figure 5(b)). Accordingly, we performed a gene enrichment analysis to identify signaling pathways contributing to the pronounced DC activation (Figure 5(c)). Interestingly, we found that the significantly regulated pathways are almost exclusively associated with type I IFN signaling (Figure 5(c)). Type I IFN signaling is known to promote the formation of IFN-stimulated gene factor 3 (ISGF3), a transcriptionally active multi-protein-complex consisting of STAT1, STAT2 and IRF9. Translocation of ISGF3 into the nucleus and subsequent binding to IFN-stimulated response elements (ISRE) drives the transcription of type I IFN and other IFN-stimulated genes (ISGs).^44^ The fact that type I IFN signaling is activated by both *H. pylori* wt and the ADP-heptose deficient ΔrfaE mutant, but more pronounced in the latter infection, is evident not only from the proteomics data (Figure 5(b)) but can also be shown by enhanced phosphorylation as well as protein expression of STAT1, STAT2 and IRF9 (Figure 5(d)). Moreover, the expression of type I IFN target genes, *IFNA2* and *ISG15*, as well as IFNα and CXCL10 secretion is significantly increased by infection with the ΔrfaE mutant compared to *H. pylori* wt infection (Figure 5(e,f)). Furthermore, it is well established that pathogen-induced type I IFN results in ISG expression but also in IFNα and IFNβ secretion resulting in an autocrine amplification loop.^45^ This amplification loop seems to be activated upon infection with the ADP-heptose-deficient mutant, as blocking of the type I IFN receptor decreases IFN signaling as well as IFN-dependent target gene expression (Supplementary Figure S4). To prove that ADP-heptose is indeed capable of dampening bacteria-induced type I IFN signaling, we treated ΔrfaE-infected DCs with ADP-heptose and analyzed type I IFN responses. As expected, the addition of ADP-heptose to ΔrfaE-infected DCs reduced the secretion of IFNα and CXCL10 (Figure 5(g)) as well as type I IFN signaling to the levels observed in *H. pylori* wt infections (Figure 5(h)). This set of data suggests a role for type I IFN in *H. pylori-*induced immune responses and identifies ADP-heptose as a negative regulator of type I IFN signaling.

We next characterized the contribution of type I IFNs to the *H. pylori-*induced DC phenotype. We found that addition of IFNα and -β increases wt-induced IL-12p70 secretion to levels obtained upon infection with the ADP-heptose-deficient mutant (Figure 5(i)). These data indicate that activation of the type I IFN signaling pathway is a pre-requisite for full-fledged DC activation in the context of *H. pylori* infection. Moreover, our flow cytometry data (Figure 5(j)) as well as the proteomics data revealed that HLA-class I family members are increased upon *H. pylori* infection (Figure 5(k)). Although type II conventional DCs used in this study are predominantly reported to be involved in activation of CD4^+^ T cells,^46^ the fact that HLA-class I expression (Figure 5(b,k)) as well as the secretion of type I IFNs is significantly enhanced in mutant-infected DCs (Figure 5(f)), prompted us to investigate the impact of ADP-heptose on CD8^+^ T cell activation. In line with our expectations, PBMCs infected with the *H. pylori* mutant lacking ADP-heptose secreted significantly higher levels of Granzyme B (GZMB) (Figure 5(l)). In addition, co-culture of mutant-infected DCs with total CD3^+^ T cells revealed a significant increase in Granzyme B production in CD8^+^ T cells in comparison to co-culture with wt-infected DCs (Figure 5(m)), suggesting that ADP-heptose dampens the *H. pylori*-induced CD8^+^ T cell activity. Accordingly, while we already showed that several immune cell subsets are recruited at a higher rate to the gastric epithelium of individuals suffering from *H. pylori* positive gastritis, we did not observe a significant increase in CD8^+^ T cells compared to *H. pylori* negative individuals (Figure 1(d)). In line with this, we do not observe a clear correlation between CD8 and Granzyme B in *H. pylori* positive gastritis, while CD8 and Granzyme B do correlate in *H. pylori* negative gastritis samples (Figure 5(n)), indicating that *H. pylori* infection might impair the effector functions of CD8^+^ T cells in the gastric mucosa. Taken together, these findings suggest that ADP-heptose prevents fully functional type I IFN signaling in *H. pylori*-infected DCs, which impairs DC activation and the subsequent CD8^+^ T cell responses.

## Discussion

In this study, we showed that the newly described PAMP, ADP-heptose, does not have the activating capacity of a bona fide PAMP in primary human DCs. Instead, ADP-heptose attenuates bacteria-induced activation of DCs and the subsequent Th1-associated T cell response. The effects of *H. pylori*-derived PAMPs such as ADP-heptose on DCs are particularly interesting in light of the finding that DCs as well as several types of lymphocytes are significantly more abundant in *H. pylori*-infected gastritis samples compared to *H. pylori* negative biopsies, which underscores the pivotal role of the immune system in the *H. pylori*-induced chronic inflammatory response.

The recognition of PAMPs by their cognate PRRs is a key feature of the innate immune system, enabling rapid anti-microbial responses to a wide variety of pathogens. While it is well established that PAMPs are potent activators of DCs, our data suggest that ADP-heptose exerts unexpected and yet unexplored effects in primary DCs. In epithelial cells, ADP-heptose is recognized by ALPK1 and induces pro-inflammatory responses. Moreover, *H. pylori* mutants devoid of ADP-heptose are less potent in triggering inflammatory responses in epithelial cells.^15,17^ The ADP-heptose receptor ALPK1 has also been described to drive inflammatory responses, as infection with the Gram-negative bacterium *Burkholderia cenocepacia* increased cytokine and chemokine expression in the lungs of wild-type mice, while these responses were compromised in *Alpk1*^−/−^ mice.^17^ Additionally, initial studies using THP-1 cells, as a surrogate for human innate immune cells, as well as primary monocytes and monocyte-derived macrophages suggested that ADP-heptose exerts similar pro-inflammatory effects on leukocytes.^47^ Taken together, these studies all point toward an immune-stimulatory role for ADP-heptose and ALPK1 signaling. On the other hand, recent reports have significantly increased our understanding of the multiple functions of ALPK1. Ryzhakov and colleagues showed that ALPK1 attenuates Th1 responses to *Helicobacter hepaticus*, by impairing the activation of innate immune cells, suggesting a suppressive role of ALPK1 in hematopoietic cells.^22^ Moreover, a recent report identifies ALPK1 signaling to be crucial for maintaining intestinal homeostasis after infection with the Gram-negative commensal *Akkersmania muciniphila*.^48^ Further elaborating on potential immunomodulatory effects of ADP-heptose/ALPK1, we show here that ADP-heptose alone is a poor activator of primary DCs. Since we could not detect ALPK1 or TIFA by whole-cell proteomics of *H. pylori* infected DCs, the poor activating capacity of ADP-heptose alone may be due to limited availability of the pathway in human primary DCs. Furthermore, in the context of a bacterial infection, it could be speculated that *H. pylori* is rapidly engulfed by DCs due to the strong phagocytic activity of DCs. This rapid uptake by the cell may locate ADP-heptose to phagosomes rather than the cytosol, making it accessible to receptors other than cytosolic ALPK1. Strikingly, bacteria devoid of ADP-heptose are even more potent in activating DCs and subsequent Th1-associated immune responses, while supplementation of ADP-heptose seems to reverse this enhanced effect on DC activation. Thus, existing data allow classification of ADP-heptose as a PAMP in the sense of a conserved microbial substance shared by different bacterial species, which is activating to epithelial cells. Regarding the activation of DCs, however, the ADP-heptose/ALPK1 axis does not seem to possess pro-inflammatory characteristics of a classical PAMP/PRR family member.

The importance of TLR2 for the activation of DCs in the context of *H. pylori* infection has been previously demonstrated by using TLR2 knock-out mice and by blocking TLR2 in human DCs. While significant decreases in IL-1β, IL-6, IL-12, IL-23, and TNFα release were observed in *H. pylori*-infected murine BM-DCs from TLR2ko mice,^49,50^ in particular the release of TNFα and GM-CSF was significantly impaired upon TLR2 blocking in human CD1c^+^ DCs.^8^ In addition, it has been shown that increased macrophage activation observed in Alpk1^−/−^ mice upon infection with *Helicobacter hepaticus* was blocked in the presence of anti-TLR2 antibodies.^22^ Along this line TLR2-blocking also impairs the activation of DCs infected with the ADP-heptose deficient mutant. In an attempt to explain the contribution of TLR2 during *H. pylori* infection, we show here that TLR2 is involved in bacterial uptake, accordingly TLR2 has already been described to mediate uptake of *Staphylococcus aureus and Pseudomonas aeruginosa*.^51,52^

Interestingly, a recent study reports that *H. pylori* has the capacity to inhibit signaling induced by the intracellular PRRs STING and RIG-I, both of which are known to induce type I IFNs.^53^ While it is known that *H. pylori* induces type I IFNs,^54,55^ Dooyema and colleagues showed that STING agonist-induced expression of ISGs is reduced upon co-infection with *H. pylori* in human organoids.^53^ In line with this finding, we report here that addition of ADP-heptose dampens the increased activation of type I IFN signaling upon infection with the ADP-heptose deficient mutant ΔrfaE to levels observed upon wt infection. This suggests that during infection with *H. pylori* wt, ADP-heptose might cause low levels of type I IFN signaling. Type I IFNs are well-established in antiviral defense, but when it comes to host susceptibility to bacterial infections, type I IFNs can have protective or deleterious effects. Recent advances reporting harmful consequences describe that type I IFNs suppress antibacterial activity upon infection with both Gram-positive and Gram-negative bacteria^56^ and exacerbate tuberculosis in a co-infection setting.^57^ Accordingly, diminished susceptibility to tuberculosis has been reported in humans harboring genetic variation in the IFNAR1 gene.^58^ In contrast, type I IFN-dependent signaling was shown to be triggered by microbiota in the skin and contributes to wound healing.^59^ Moreover, STING-mediated type I IFN responses correlate with protection against *Streptococcus pyogenes* infection.^60^ In the context of *H. pylori*, a protective role was suggested as well, in that IFNAR-deficient mice exhibit increased susceptibility to *H. pylori*.^61^

It has been recently reported that CD8^+^ T cells play a crucial role in controlling *H. pylori* infection^62^ and type I IFNs are important drivers of effective expansion and granzyme B production by CD8^+^ T cells in the context of viral infections. ^63–65^ Yet, upon chronic infection with *H. pylori*, CD8^+^ T cells lose their resident memory phenotype and seem to be replaced by CD4^+^ T cells.^62,66^ Although conventional DCs used in this study, are not known as the main activators of CD8^+^ T cells, recently a novel DC phenotype has been described, termed ISG^+^ DC, which are cDC2s characterized by increased expression of IFN stimulated genes.^67^ In a tumor context, MHC class I dressed ISG^+^ DCs exert enhanced capacity to stimulate CD8^+^ T cells, resulting in effective anti-tumor T cell immunity.^67^ Moreover, DCs from *H. pylori*-infected mice are less potent in promoting tumor-specific CD8^+^ T cell proliferation, resulting in impaired efficacy of cancer immunotherapies.^68^ In light of these studies, the finding that ADP-heptose attenuates type I IFN signaling upon *H. pylori* infection is of great interest, as it suggests a potential mode of action by which *H. pylori* mitigates CD8^+^ T cell responses. Further studies are necessary to identify whether the ADP-heptose driven attenuation of type I IFN responses mediates the effects on CD8^+^ T cell responses and to clarify whether ADP-heptose affects host susceptibility to *H. pylori*.

Taken together, our study findings emphasize the role of DCs in the gastric mucosa and show for the first time that ADP-heptose, despite its potent pro-inflammatory functions in epithelial cells, does not act as a bona fide PAMP in human primary DCs. Rather, ADP-heptose attenuates bacteria-induced immune responses by disrupting the positive feedback loop enforced by type I IFN signaling, thus dampening DC activation and the subsequent T cell response.

## Supplementary Material

Supplemental Material
