# Supplementary material for: ADP-heptose attenuates Helicobacter pylori-induced dendritic cell activation
Source: Gut Microbes. 2024 Sep 17;16(1):2402543. doi: 10.1080/19490976.2024.2402543 (PMC11409497; doi:10.1080/19490976.2024.2402543)
Supplement: Supplemental Material [file KGMI_A_2402543_SM5102.zip › Supplementary_Figures.docx]

# Supplementary Figures 1-4


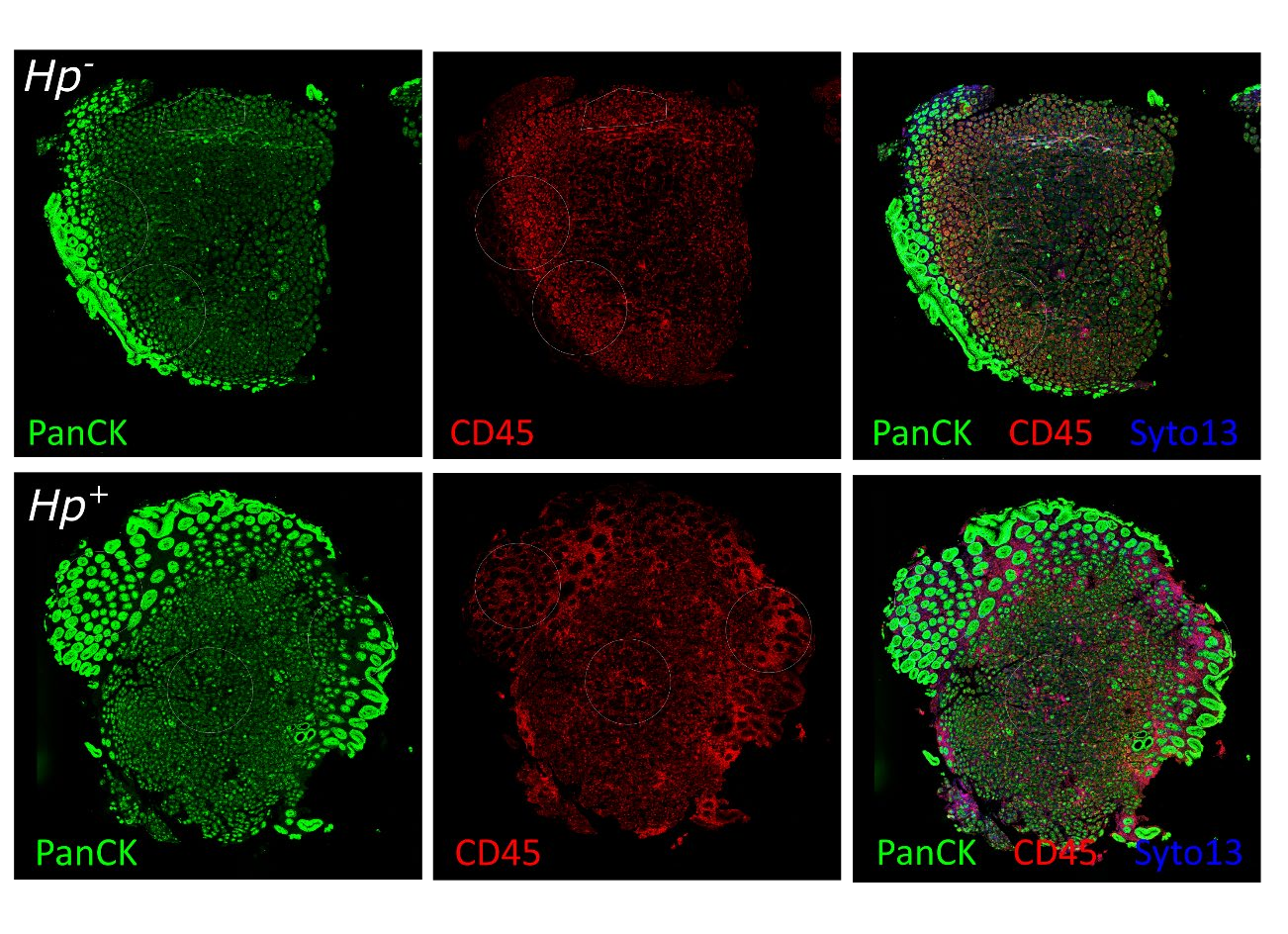


**Supplementary Figure 1 Single staining of gastric biopsies of *H. pylori* positive and negative gastritis samples.** Fluorescence staining of FFPE sections of gastric biopsies from *H. pylori* negative (Hp^-^) and *H. pylori* positive (Hp^+^) gastritis patients using PanCK for epithelial cells (green), CD45 to identify immune cells (red) and Syto13 nuclear staining (blue). One out of three samples per group is shown.


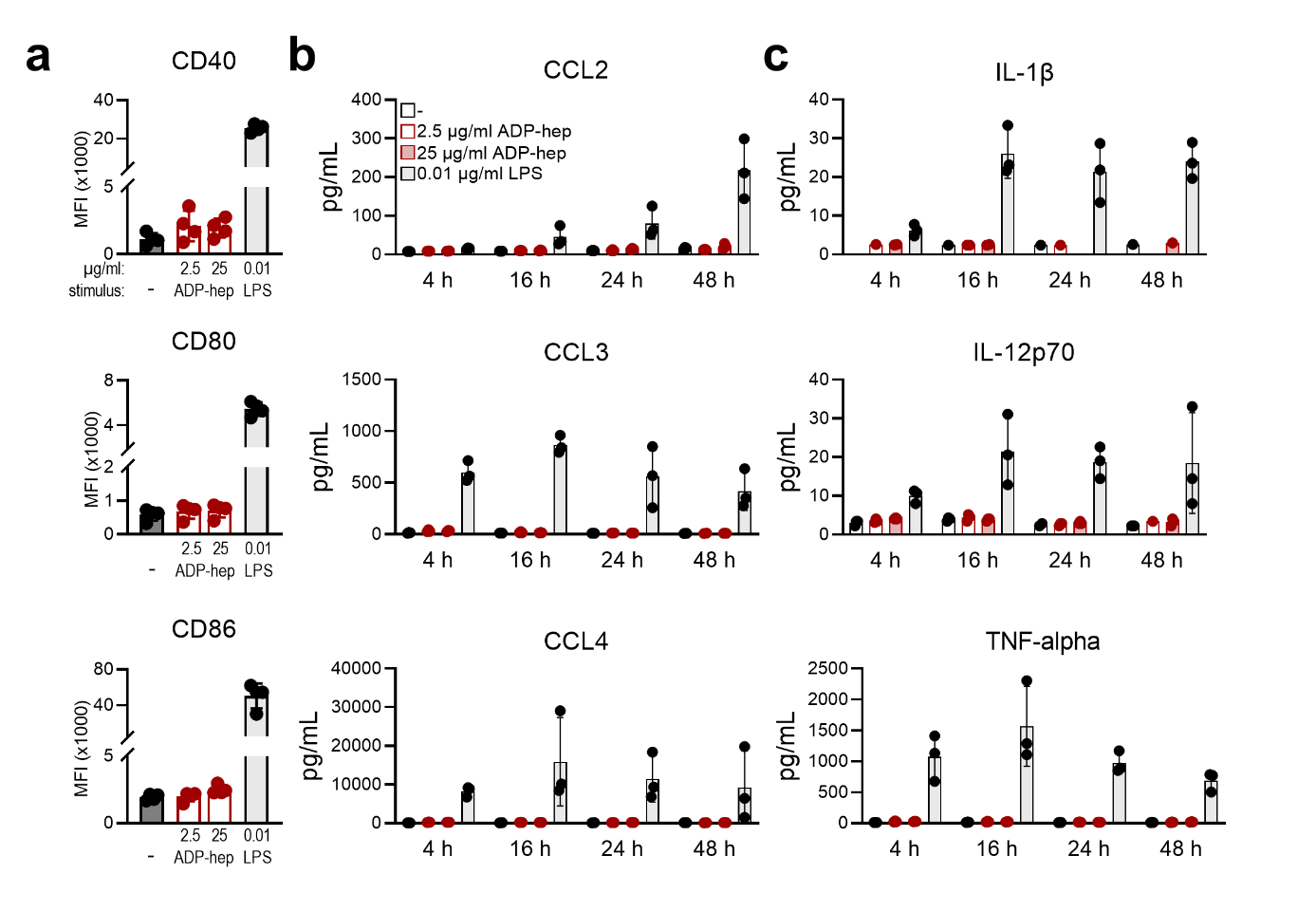


# Supplementary Figure 2 ADP-heptose is not as efficient as *E. coli* LPS in activating DCs.

Human CD1c^+^ DCs were isolated from peripheral blood of healthy donors by magnetic cell sorting and incubated with ADP-heptose (2.5 or 25 µg/mL) or *E. coli* LPS (10 ng/mL) for 16 h **(a)** or the indicated time points ranging from 4 to 48 h **(b/c)**. Surface marker expression **(a)** or chemokine and cytokine secretion **(b/c)** was analyzed by flow cytometry or multiplex assay, respectively. Bars indicate mean±SD of three independent donors, dots represent individual donors.


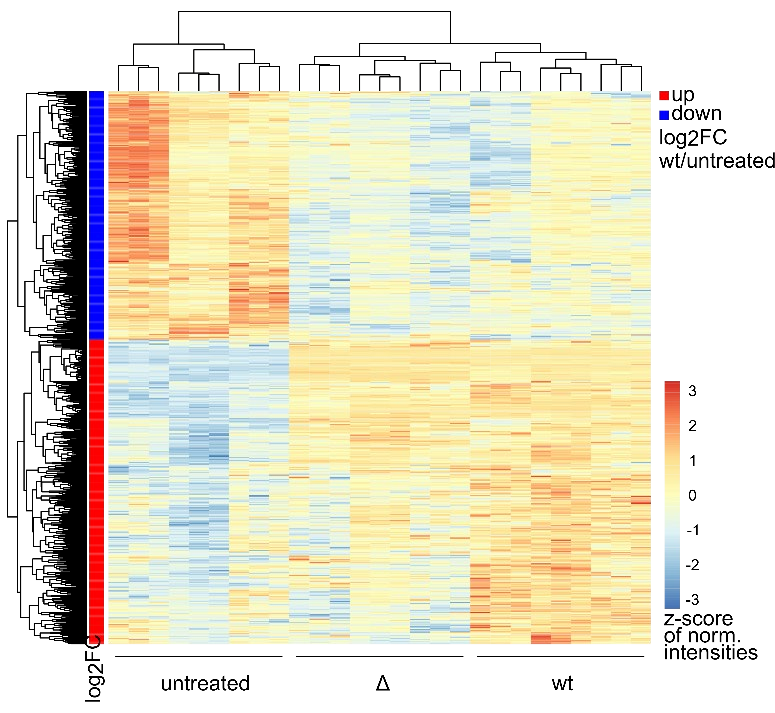


**Supplementary Figure 3 Protein expression in human primary DCs in response to *H. pylori* (wt) or the ADP-heptose devoid mutant (ΔrfaE)**. Heatmap showing z-scores of normalized intensities for differentially expressed proteins in DCs upon infection with *H. pylori* (wt), the ADP-heptose devoid mutant (Δ) or untreated (n=3). Red/blue annotation for direction of change indicates up/down regulation of differentially expressed proteins present in the comparison of wt vs. untreated, respectively.


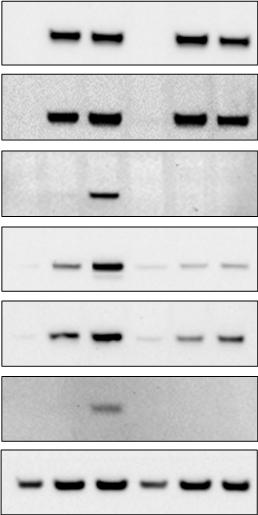
1.5

mRNA

expression

| **a** *H. pylori*: | - wt | Δ | - | wt | Δ **b** |
| --- | --- | --- | --- | --- | --- |
| αIFNAR:  pSTAT1 | - - | - | + | + | + |
| STAT1 |  |  |  |  | ative |

1.0

0.5

rel

0.0

*ISG15*


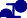

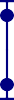

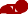


pSTAT2

*H. pylori*: - wt Δ - wt Δ αIFNAR: - - - + + +

STAT2 IRF9 ISG15

0.15

relative mRNA expression

0.10

0.05

0.00

*CXCL10*


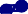


β-Actin

*H. pylori*: - wt Δ - wt Δ

αIFNAR: - - - + + +

**Supplementary Figure 4 ADP-heptose devoid *H. pylori* mutant drives an autocrine type I IFN loop.** Blocking of the IFN-Alpha/Beta Receptor Chain 2 (1 or 5 µg/ml) was performed 20 min prior to infection. Intracellular type I IFN signaling **(a)**, target gene expression **(b)** were monitored 16 h post- infection with *H. pylori* (wt) or the ADP-heptose devoid mutant (Δ). One representative donor out of two is shown **(a)**. **(b)** Bars indicate mean±SD of two donors, dots represent individual donors.
